# Mechanistic research on the vestibular-hippocampal pathway in neurodegenerative diseases: an integrative perspective from molecular to behavioral levels

**DOI:** 10.3389/fnins.2026.1779268

**Published:** 2026-04-07

**Authors:** Dingyuan Dai, Junyan Chen, Xiang Guo, Jiabing Sun, Haijin Yi

**Affiliations:** 1 Department of Otorhinolaryngology-Head and Neck Surgery, Beijing Tsinghua Changgung Hospital, School of Clinical Medicine, Tsinghua University, Beijing, China; 2 School of Clinical Medicine, Tsinghua University, Beijing, China; 3 Department of Otorhinolaryngology-Head and Neck Surgery, Zhuhai People’s Hospital, Zhuhai, China

**Keywords:** vestibular system, hippocampus, neurodegenerative disease, cognition, spatial memory

## Abstract

This paper systematically reviews the pivotal role and bidirectional regulatory mechanisms of the Vestibular-hippocampal pathway in the onset and progression of neurodegenerative diseases (such as Alzheimer’s disease), focusing on the common comorbidity of vestibular dysfunction and cognitive decline. Evidence spanning molecular to behavioral levels indicates that vestibular signal loss can induce hippocampal atrophy and spatial memory impairment through neuroinflammation, impaired synaptic plasticity, and disrupted theta rhythms. Conversely, hippocampal degeneration further impairs vestibular information integration, creating a vicious cycle. Intervention approaches such as vestibular rehabilitation, cognitive training, and neurostimulation show potential for slowing co-morbidity progression. Future research should focus on developing animal models simulating vestibular-neurodegenerative co-morbidity, conducting longitudinal clinical validation using multimodal imaging and electrophysiology techniques, and optimizing neuromodulation strategies and targeted molecular interventions to advance this mechanism toward early diagnosis and precision treatment.

## 1 Introduction

Neurodegenerative diseases (NDDs) constitute a group of disorders characterized by progressive loss neuroinflammatory processes (Li et al., 2023). With the accelerating pace of global population aging, neurodegenerative diseases such as Alzheimer’s disease (AD) and Parkinson’s disease (PD) have become a global public health crisis. As of 2023, over 55 million people worldwide have been diagnosed with dementia. This clinical syndrome typically manifests in the late stages of Alzheimer’s disease, Parkinson’s disease dementia, and various other neurodegenerative and systemic diseases. The total number of patients is projected to rise to 139 million by 2050 (Mecca and van Dyck, 2021). Despite significant advances in elucidating molecular mechanisms (e.g., β-amyloid deposition, tau hyperphosphorylation) and identifying genetic risk factors (e.g., APOE ε4, PSEN mutations), therapeutic approaches targeting these core pathological proteins have repeatedly failed in clinical trials (Formolo et al., 2023; Si et al., 2023; Mojarad-Jabali and Roh, 2025; Lozupone et al., 2024). Traditional research approaches may have overly focused on the single-neuron level while overlooking systemic interactions, particularly the influence of sensory input systems (e.g., the vestibular system) on cognitive hubs (e.g., the hippocampus) (Guo et al., 2024). Recent studies indicate that sensory impairments (e.g., hyposmia, vestibular dysfunction) often precede typical cognitive symptoms by several years (Bathini et al., 2019; Cai et al., 2023). These abnormalities may serve as early warning signals for neurodegenerative disease, offering potential intervention targets and creating a critical window for developing therapeutic strategies.

The hippocampus is the core region of the brain responsible for spatial memory and navigation (Harvey et al., 2018), while the vestibular system plays a crucial role in balance and spatial orientation by detecting head movements and gravity (Besnard et al., 2018; Beiza-Canelo et al., 2023). These two systems do not operate in isolation; research indicates that the vestibular system is closely connected to the hippocampus via neural pathways, forming the “Vestibular-hippocampal pathway” that influences cognitive functions (Guo et al., 2024; Harvey et al., 2018; Hitier et al., 2014; Smith, 1997). Specifically, vestibular input is essential for maintaining the activity of the hippocampus’s place cells and head-direction cells, directly contributing to the stability of spatial memory (Harvey et al., 2018). However, vestibular dysfunction (such as bilateral vestibular loss) may lead to impaired neurogenesis and synaptic plasticity in the hippocampus, triggering neuronal atrophy and cell death, which in turn induces cognitive impairment (Guo et al., 2024; Zhu et al., 2024). In neurodegenerative diseases (such as late-stage Alzheimer’s disease), this pathway may exacerbate disease progression by mediating spatial disorientation and memory deficits (Zhou et al., 2024). Therefore, exploring the role of the Vestibular-hippocampal pathway not only aids in understanding the mechanisms of neurodegenerative diseases but may also offer new perspectives for the diagnosis and treatment of cognitive impairments.

Given the critical role of the Vestibular-hippocampal pathway in maintaining cognitive function and linking vestibular dysfunction to core pathological changes in neurodegenerative diseases, this review aims to provide an integrated perspective, systematically elucidating the pathway’s role in the pathomechanisms of neurodegenerative diseases and potential intervention strategies. This paper synthesizes evidence spanning from microscopic molecular mechanisms to macroscopic behavioral manifestations, aiming to elucidate the dysregulation mechanisms of Vestibular-hippocampal functional connectivity in disease. It further explores intervention strategies targeting this pathway and their translational potential, with the goal of offering novel insights for the early diagnosis and treatment of neurodegenerative diseases.

## 2 The vestibular system and hippocampal structure

### 2.1 Anatomy and function of the vestibular system

The vestibular system comprises peripheral and central components: (1) The peripheral portion primarily refers to the sensory organs located within the inner ear, including the utricle, saccule, and semicircular canals. These organs detect head orientation, acceleration, and gravitational information, converting physical motion into neural signals. Specifically, the utricle and saccule (collectively termed the otolith system) primarily sense linear acceleration and gravity (e.g., head tilt), while the semicircular canals detect angular acceleration (e.g., rotational motion). Signals detected by the peripheral components are transmitted via the vestibular nerve to the vestibular nucleus (VN) in the brainstem (Besnard et al., 2018; Smith et al., 2024). (2) The central system comprises a distributed neural network extending from the brainstem to the cortex, including the vestibular nuclei (VN), vestibular regions of the cerebellum (e.g., flocculus, vermis, and nodulus/flocculonodular complex), thalamus, and cortical areas (e.g., vestibular cortex). These regions not only process signals from peripheral vestibular organs but also integrate multimodal information from vision, proprioception, and other sources to support complex cognitive and reflex functions (e.g., spatial orientation and postural control) (Cullen, 2023). The entire signal transmission pathway can be summarized as a linear sequence: peripheral vestibular organs → brainstem vestibular nuclei (VN) → cerebellum/thalamus → cortical or spinal cord output structures. This pathway is highly modifiable during development and compensation (Lai et al., 2023; Goldblatt et al., 2023). The vestibular system plays a central role in cognitive functions such as balance reflexes, spatial navigation, and spatial memory (Guo et al., 2024; Besnard et al., 2018; Wiboonsaksakul et al., 2024).

However, the vestibular system’s function extends far beyond that of a passive linear signal relay station. Its core role lies in serving as a dynamic multisensory integration hub, continuously calibrating and optimizing information streams from bilateral vestibular, visual, auditory, and somatosensory inputs to construct stable spatial perception and behavioral output (Ponzo et al., 2019; Kaliuzhna et al., 2016). This integrative capacity proves particularly critical during aging and neurodegenerative diseases, where its disruption is recognized as a key mechanism linking sensory deficits to cognitive decline.

The dynamic equilibrium of bilateral vestibular input forms the physiological basis for spatial perception. Under physiological conditions, the resting discharge rates of bilateral vestibular organs maintain relative symmetry, providing a stable baseline signal to the brainstem vestibular nuclei (Newlands et al., 2018; Laurens et al., 2017; Robinson, 2022). When head motion occurs, this equilibrium is disrupted. Head movement causes an imbalance in bilateral afferent signals (Newlands et al., 2018; Laurens et al., 2017; Robinson, 2022). The central nervous system decodes these interaural signal differences to precisely calculate the angular velocity and linear acceleration of the head, thereby establishing a perceptual reference frame for self-motion and spatial relationships (Newlands et al., 2018; Laurens et al., 2017; Robinson, 2022). Unilateral vestibular loss (UVL) immediately disrupts this equilibrium, causing acute syndromes such as spontaneous nystagmus, postural asymmetry, and head tilt (Keshavarzi et al., 2022). More critically, this imbalance in peripheral input triggers central compensatory reorganization involving cross-modal plasticity changes, including enhanced visual and somatosensory compensation (Keshavarzi et al., 2022). Research indicates that even after acute motor symptoms are compensated, alterations in hippocampal neuronal activation patterns and synaptic plasticity impairments induced by UVL persist, leading to long-term deficits in spatial reference memory and working memory (Zhou et al., 2024). This suggests that imbalance in bilateral vestibular input not only affects immediate postural reflexes but may also “upstream” disrupt the speed and accuracy of hippocampal-dependent cognitive information processing through abnormal neural activity patterns.

Secondly, the vestibular and visual systems exhibit functional coupling, jointly forming the core of self-motion perception. Although the angular head velocity (AHV) signal in the retrosplenial cortex primarily relies on vestibular input, visual input significantly enhances its coding gain and signal-to-noise ratio, thereby optimizing the accuracy of self-turn perception during navigation (Keshavarzi et al., 2022). This vestibular-visual integration is crucial for precise spatial orientation and path integration in complex environments. During aging, functional decline in visual motion processing pathways (e.g., abnormal visuomotor responses), compounded by increased vestibular signal noise, impairs multisensory integration’s optimization capacity in sensory conflict scenarios (Kenney et al., 2021; Ramkhalawansingh et al., 2018). This results in failure to achieve Bayesian optimal integration and erroneous reliance on unreliable visual cues (Ramkhalawansingh et al., 2018). The concomitant reduction in integration efficiency, coupled with neural processing delays and diminished brain network plasticity, collectively increases spatial cognitive load. Manifestations include unstable postural control and heightened spatial orientation errors.

Additionally, the functional integration of vestibular information with the auditory spatial localization system constitutes another key mechanism. The vestibular system provides a head-centered reference frame, which serves as the foundation for the auditory system to analyze binaural time and intensity differences, thereby determining the direction of sound sources (Wang et al., 2025). Aging is often accompanied by age-related hearing loss (ARHL), which leads to decompensation in the dorsolateral prefrontal cortex (DLPFC) during noisy environments. This manifests as weakened theta-band neural oscillations and reduced connectivity within the frontotemporal network, thereby impairing the top-down regulatory function of auditory-motor integration (Wang et al., 2025).

In summary, as the core hub for multisensory integration, dysfunction of the vestibular system amplifies and exacerbates multisensory desynchronization. This leads to spatial cognitive disorientation, impaired postural control, and may accelerate neurodegenerative pathology by increasing abnormal metabolic load in cognitive brain regions such as the hippocampus (Roytman et al., 2025). Therefore, when investigating the role of the Vestibular-hippocampal pathway in neurodegenerative diseases, it must be situated within a dynamic framework of multisensory interaction. This approach examines how sensory integration imbalance acts as an upstream driver, influencing hippocampus-dependent memory and navigation functions.

### 2.2 The hippocampal structure and its role in cognition

The hippocampus (HPC) is located within the medial temporal lobe (MTL) and, together with the entorhinal cortex, forms the hippocampal-entorhinal complex. This region is a key brain area for learning, memory, and spatial cognition (Kobro-Flatmoen et al., 2021; Lang et al., 2024). The internal structure of the hippocampus exhibits high heterogeneity, primarily comprising multiple functionally distinct subregions including the dentate gyrus (DG), CA1 region, CA2 region, CA3 region, and subiculum (Sub). These subregions play different roles within neural circuits, collectively supporting the hippocampus’s core functions in learning and memory, spatial navigation, and emotional and stress responses (Harvey et al., 2018; Beiza-Canelo et al., 2023; Brandt and Dieterich, 2017). The entorhinal cortex, serving as a crucial hub connecting the neocortex to the hippocampus, integrates and transmits spatial information to the hippocampus (Smith et al., 2024).

Place cells, enriched in the hippocampal CA1–CA3 regions, are a key component of the brain’s spatial memory system. Their primary function is to encode spatial information in the environment by firing at specific locations, thereby forming anchor points for cognitive maps (Bhasin and Nair, 2022; Knudsen and Wallis, 2021). Head-direction cells exhibit a distributed pattern throughout the brain, with core nodes located in the anterior thalamus and medial entorhinal cortex (MEC), extending into parahippocampal regions. They encode the direction of the animal’s head orientation, providing directional reference for spatial navigation (Xu et al., 2019). Grid cells, primarily located in the MEC, form a spatial coordinate system for precise navigation through their regular hexagonal firing patterns (Xu et al., 2022). Position cells and grid cells synchronize their rhythmic firing via bidirectional synaptic connections. In collaboration with spatial neurons like head-direction cells, they form a dynamic functional network that integrates spatial information, ultimately constructing a cognitive map supporting precise localization and navigation (Long and Zhang, 2021; Aronov et al., 2017). Notably, the vestibular system plays a crucial regulatory role in this process. Vestibular information not only maintains the stability of head-direction cell signals but also significantly influences the spatial reliability of hippocampal place coding (Lai et al., 2023). Furthermore, hippocampal function exhibits distinct differentiation along its longitudinal axis (dorsoventral or anteroposterior axis): the dorsal (posterior) hippocampus primarily engages in spatial learning and memory, while the ventral (anterior) hippocampus is more involved in regulating emotional and anxiety-related behaviors (Besnard et al., 2018; Beiza-Canelo et al., 2023). Hippocampal dysfunction is closely associated with epilepsy and mood disorders (Liu et al., 2026), and impaired neurogenesis is recognized as a key mechanism underlying cognitive deficits in various central nervous system diseases. During normal aging, the hippocampus undergoes mild degenerative changes; however, in neurodegenerative diseases such as Alzheimer’s disease (AD), it frequently exhibits marked atrophy and functional impairment (Harvey et al., 2018).

### 2.3 Vestibular-hippocampal pathway

The vestibular system, as a key sensory system for perceiving gravity and spatial motion, plays a crucial role in maintaining spatial memory and cognitive functions through its signal inputs (Brandt and Dieterich, 2017). The vestibular system and hippocampus are interconnected anatomically and functionally, jointly forming the Vestibular-hippocampal pathway (VHP)—a critical circuit for spatial information processing (Hitier et al., 2014; Smith, 1997). This pathway is not a single direct projection but comprises multiple parallel, polysynaptic pathways responsible for conveying vestibular information to the hippocampus. This pathway system is regarded as the essential neural foundation for spatial cognition (Hitier et al., 2014; Smith, 1997; Yun et al., 2025; Smith et al., 2025; Adel Ghahraman et al., 2016).

Vestibular signals primarily transmit to the hippocampus via the following core pathways: (1) The vestibular-thalamic-cortical pathway follows this route: Vestibular nucleus complex (VNC) → Thalamic nuclei (primarily the ventral posterolateral thalamic nucleus) → Parietal cortex → Entorhinal cortex → Hippocampus (Hitier et al., 2014; Smith, 1997; Smith et al., 2025; Zwergal et al., 2024). This pathway serves as the primary route for ascending vestibular information to higher cognitive regions, with functions including: ① Conveys integrated spatial position and head movement direction information; ② Participates in constructing environmental spatial cognitive maps; ③ Mediates self-motion cue-based spatial navigation behaviors (Smith et al., 2025; Shinder and Taube, 2010; Biazoli et al., 2006; Stackman et al., 2002). (2) Theta Rhythm Pathway: Vestibular nucleus complex (VNC) → Pedunculopontine tegmental nucleus (PPTg) → Supramammillary nucleus (SUM) → Posterior hypothalamic nucleus → Medial septum → hippocampus (Smith et al., 2025; Zwergal et al., 2024; Aitken et al., 2018). The core mechanism of this pathway is that vestibular stimulation may increase acetylcholine release from the medial septum to the hippocampus, where acetylcholine drives hippocampal theta rhythms and modulates synaptic plasticity. Its functions include: ① Maintaining spatial memory encoding and consolidation by driving hippocampal theta rhythms (4–12 Hz); vestibular dysfunction impairs spatial memory and theta rhythms (Smith et al., 2025; Aitken et al., 2017; Tai et al., 2012). ② Coordinating firing patterns of place cells and grid cells to support spatial navigation and representation (Zwergal et al., 2024; Tai et al., 2012; Neo et al., 2012; Russell et al., 2006; Smith et al., 2005). ③ Enhancing synaptic plasticity via cholinergic mechanisms, potentially mediating vestibular-related learning and memory processes (Tai et al., 2012; Smith et al., 2020; Tai and Leung, 2012; Besnard et al., 2012). (3) Head Direction Pathway, with the following pathway: vestibular nucleus complex (VNC) → dorsal tegmental nucleus (DTN) → lateral mammillary nucleus (LMN) → anterodorsal thalamic nucleus (ADN) → postsubiculum (PoS) → entorhinal cortex (EC) / hippocampus (Hitier et al., 2014; Smith et al., 2005; Hu et al., 2025). This pathway constitutes the core hardware of the brain’s internal “compass” system. Its function is to encode the animal’s current head orientation, providing an intrinsic reference frame for spatial navigation and ensuring precise orientation within the environment. This function is primarily achieved through the directionally selective firing of HD cells, supporting a compass-like sense of direction (Xu et al., 2019; Zirkelbach and Herz, 2019; Mehlman et al., 2019). (4) Vestibulo-Cerebellar-Cortical Pathway: Vestibular Nucleus Complex (VNC) → Cerebellum → VlN (ventrolateral nucleus) / VP (ventropostral nucleus) → Parietal Cortex → EC (entorhinal cortex) / Hippocampus (Guo et al., 2024; Huang et al., 2023; Leandri et al., 2015). Its functions include: ① Neural basis for spatial learning and navigation: This pathway integrates vestibular motion signals with visual/proprioceptive inputs, providing a computational framework for spatial learning and navigation (Guo et al., 2024; Huang et al., 2023); ② Interaction between vestibular signals and cortical cognitive functions: This pathway transmits vestibular information to parietal cortex (e.g., posterior parietal cortex, PPC), participating in spatial working memory and attentional control (Leandri et al., 2015; Noel and Angelaki, 2022); ③ Indirect regulation of hippocampal spatial representations: Through parietal-entorhinal cortex projections, this pathway indirectly influences the spatial coding of hippocampal place cells and grid cells (Guo et al., 2024; Smith et al., 2025).

## 3 Bidirectional deterioration mechanism of the vestibular-hippocampal pathway in neurodegenerative diseases

**Figure 1:**
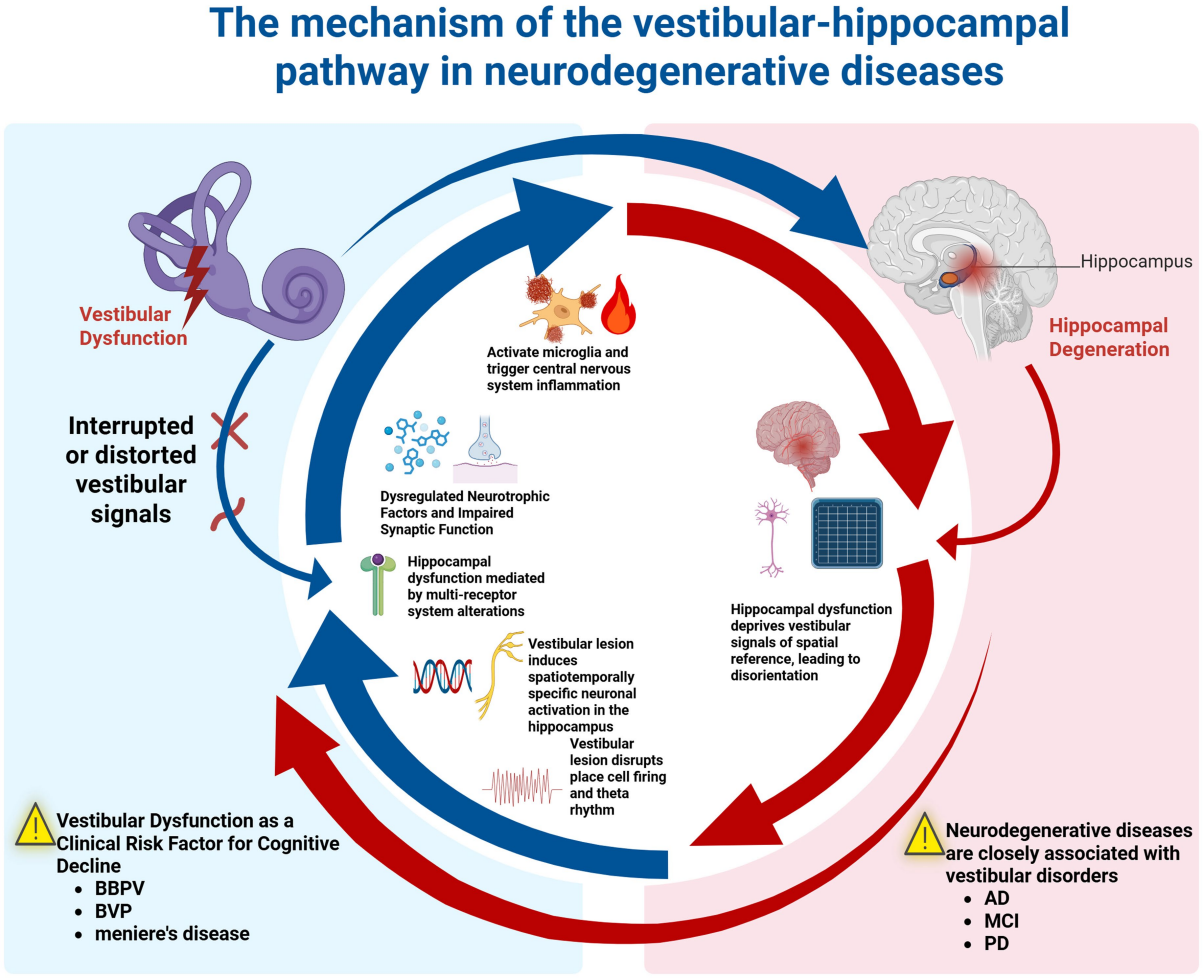
The mechanism of the vestibular-hippocampal pathway in neurodegenerative diseases: this schematic illustrates the bidirectional vicious cycle of the vestibular-hippocampal pathway in neurodegenerative diseases. Blue arrows indicate that vestibular dysfunction induces neuroinflammation, disrupts synaptic function and neural rhythms, leading to hippocampal damage and spatial cognitive decline. Red arrows indicate that hippocampal degeneration, in turn, weakens the spatial integration of vestibular signals, exacerbating orientation disorders. These two processes mutually reinforce each other, forming the core mechanism of co-morbidity between cognitive and vestibular symptoms.

Extensive evidence from animal models and clinical studies indicates that the relationship between the vestibular system and the hippocampus is not a unidirectional functional dependency, but rather a tightly coupled, bidirectionally regulated functional pathway (Hitier et al., 2014; Yun et al., 2025; Smith et al., 2025). The vestibular system provides the self-motion information essential for the hippocampus to construct spatial memory, while the hippocampus integrates and interprets this information to guide navigation and behavior (Harvey et al., 2018; Besnard et al., 2018). Dysfunction in one system triggers degenerative changes in the other, ultimately forming a self-reinforcing vicious cycle that accelerates the progression of neurodegenerative diseases (Guo et al., 2024; Bosmans et al., 2024; Kamil et al., 2018) (Figure 1).

### 3.1 Effects of vestibular system damage on hippocampal structure and function

Vestibular dysfunction directly induces significant alterations in the hippocampus’s structure, electrophysiology, and functional molecular expression by reducing or distorting afferent signals of self-motion transmitted to the central nervous system, thereby impairing its spatial cognitive functions (Adel Ghahraman et al., 2016; Aitken et al., 2018; Kamil et al., 2018).

(1) Molecular-level mechanisms (based on cellular and animal studies): ① Neuroinflammation and glial cell activation: Animal models such as unilateral vestibular nerve section (UVN) demonstrate that vestibular injury activates microglia, triggering central neuroinflammation (El Mahmoudi et al., 2022; Rastoldo et al., 2021). This inflammatory response extends beyond brainstem vestibular nuclei to ascend and affect structures like the hippocampus (El Mahmoudi et al., 2022; Harbin et al., 2023; Zhang L. et al., 2022). Activated microglia release pro-inflammatory cytokines (e.g., IL-1β, TNF-*α*), which impair synaptic plasticity and may exacerbate Alzheimer’s disease (AD)-associated Aβ and tau pathology (Ebrahimi et al., 2025). ② Neurotrophic Factors and Altered Synaptic Plasticity: Studies in bilateral vestibular leavage (BVL) rat models indicate that signaling pathways involving brain-derived neurotrophic factor (BDNF) and its receptor TrkB—key molecules for synaptic plasticity and learning/memory in the hippocampus—may be disrupted (Koriath et al., 2025). Research suggests BDNF/TrkB signaling is critical for vestibular compensation, accelerating functional recovery by promoting neural plasticity (Mao et al., 2021). The absence of vestibular input may weaken hippocampal neurotrophic support, thereby impairing synaptic function. Concurrently, altered expression of molecules such as neuronal nitric oxide synthase (nNOS) and arginase I/II suggests damage to the fundamental basis of synaptic plasticity and signal transduction (Liu et al., 2004; Liu et al., 2003a; Zheng et al., 2001). These molecular-level alterations may precede macrostructural changes and form the foundation for subsequent functional deficits. ③ Alterations in Receptor and Neurotransmitter Systems: The absence of vestibular input significantly disrupts the hippocampal neurochemical microenvironment, impairing synaptic plasticity and spatial memory function through changes in multiple receptor and neurotransmitter systems. Animal studies demonstrate that unilateral vestibular deafferentation (UVD) leads to long-term downregulation of NMDA receptor subunits (NR1, NR2A) in hippocampal CA2/3 regions (Liu et al., 2003b). Conversely, in unilateral vestibular neurectomy (UVN) models, the GluN2B subunit of NMDA receptors in CA3 exhibits long-term upregulation, closely associated with spatial memory deficits (El Mahmoudi et al., 2023). Conversely, bilateral vestibular loss (BVL) exhibits significantly increased NMDA receptor density with reduced affinity, accompanied by enhanced LTP and E-S, suggesting that absent vestibular input may disrupt memory encoding through non-specific amplification of glutamatergic synaptic transmission (Besnard et al., 2012; Truchet et al., 2019). Beyond the glutamatergic system, other modulatory receptors are affected: BVL rats exhibit impaired hippocampal endocannabinoid systems, manifested by significant downregulation of CB1 receptors in the CA3 region, which weakens synaptic plasticity and neuroprotective functions (Baek et al., 2011). Simultaneously, BVL induced sustained downregulation of M1 muscarinic acetylcholine receptors in CA1, CA2/3, and the dentate gyrus (significantly reduced after 30 days, *p* ≤ 0.0001). This alteration may not only exacerbate glutamatergic dysfunction coupled with NMDA receptor function but also impede LTP induction by weakening ERK/MAPK pathway regulation (Aitken et al., 2016). Furthermore, the UVD model revealed that vestibular input loss also downregulates cytoplasmic glucocorticoid receptor (GR) expression in hippocampal CA1, suggesting potential further attenuation of neuroplasticity through stress response system modulation (Lindsay et al., 2005). Collectively, these findings demonstrate that vestibular dysfunction disrupts hippocampal synaptic plasticity via multi-receptor molecular alterations, providing a neurochemical basis for impaired spatial memory and learning. ④ Neuronal Activity Alterations: Unilateral labyrinthectomy (UL) studies reveal that vestibular injury induces temporally specific neuronal activation in the hippocampal dentate gyrus (DG). Immunofluorescence staining demonstrates a significant increase in c-Fos-positive neurons within the DG at 8 h, 5 days, and 7 days post-UL surgery, indicating sustained enhanced neuronal activity. This activation exhibits distinct spatiotemporal dynamics: initial concentration in the polymorphic layer followed by gradual expansion into the granular cell layer, reflecting differential engagement of distinct cell subpopulations in injury response and compensation. This finding further suggests that vestibular input deprivation triggers early transcriptional responses in hippocampal neurons, potentially contributing to cognitive impairment mechanisms by regulating gene expression associated with synaptic function and neural plasticity (Zhou et al., 2024).
(2) Electrophysiological and Circuit Levels: Vestibular lesions exert rapid and persistent disruptive effects on hippocampal neural activity at both electrophysiological and circuit levels. Bilateral vestibular lesioning (BVL) immediately induces spatial fragmentation, loss of specificity, and temporal instability in hippocampal place cells (Smith et al., 2015). Concurrently, the absence of vestibular input correlates closely with reduced power and diminished regularity of hippocampal theta rhythms. Studies in the BVL model demonstrate that vestibular damage significantly amplifies the power of somatosensory-induced theta rhythm type 2 (3–6 Hz), accompanied by increased cholinergic neuron numbers in the pontine tegmental nucleus (PPTg) and altered hippocampal acetylcholine release levels. This suggests the vestibular system may influence hippocampal rhythmic activity by regulating the cholinergic “theta-generating pathway” (Aitken et al., 2017). Systemic studies further confirmed that BVL rats exhibited reduced hippocampal theta rhythm power (approximately 61.5%), slowed frequency (7.79 Hz → 7.42 Hz), and diminished rhythmicity at 60 days post-surgery and beyond. These alterations were independent of locomotor speed or stress levels, indicating that vestibular input exerts a specific regulatory role in maintaining normal theta oscillations (Russell et al., 2006). Given the critical role of theta rhythms in encoding and consolidating spatial memory, their disruption not only impairs neural network coordination but may also exacerbate spatial cognitive deficits by interfering with the phase encoding of place cells.
(3) Structural and Behavioral Levels: The aforementioned alterations at the molecular and electrophysiological levels ultimately translate into observable structural atrophy and behavioral deficits. Clinical imaging studies confirm that in 10 patients (4 females, 6 males; mean age 38.0 ± 6.7 years) who underwent bilateral vestibular nerve resection for neurofibromatosis type 2 (5–10 years post-surgery), resulting in chronic bilateral vestibular loss (BVL), showed a significant 16.9% reduction in hippocampal volume compared to a gender-, age-, and education-matched healthy control group (10 subjects, mean age 38.7 ± 5.4 years) (Aitken et al., 2016; Allen et al., 2007; Kremmyda et al., 2016). This atrophy was significantly correlated with spatial memory and navigation deficits observed in the virtual Morris water maze (vMWM) task, such as reduced platform quadrant search time and increased initial directional error (Aitken et al., 2016; Allen et al., 2007; Kremmyda et al., 2016). Clinical observations further indicate that various vestibular disorders are closely associated with cognitive risk: - Patients with advanced Meniere’s disease (MD stages 3–4) exhibit severe endolymphatic hydrops and significant hippocampal atrophy, with a positive correlation between the two; they also demonstrate marked deficits in attention, memory, and executive function (Lin et al., 2025; Jian et al., 2024; Zachou and Bronstein, 2023). Patients with benign paroxysmal positional vertigo (BPPV) may experience residual dizziness after repositioning, with resting-state fMRI revealing abnormal functional connectivity between hippocampal subregions and multiple cognition-related brain areas; patients with bilateral vestibular lesions (BVP) exhibit persistent spatial navigation impairments, with imaging confirming significant hippocampal volume reduction, and the degree of atrophy directly correlating with cognitive deficits (Kremmyda et al., 2016; Lee et al., 2023; Brandt et al., 2005). A cross-sectional study by Bosmans et al. (2022) revealed that after strictly controlling for age, gender, and hearing status, patients with bilateral vestibular lesions exhibited clinically significant overall cognitive decline (lower RBANS-H total scores), with the most pronounced impairments in immediate memory, visuospatial cognition, and attention (Gommeren et al., 2023; Bosmans et al., 2022). Although the study design could not elucidate the specific temporal trajectory of cognitive decline, the results suggest this decline is more likely associated with neurophysiological alterations caused by the absence of vestibular input itself, rather than secondary to psychosocial adaptation factors. Researchers further compared the cognitive impairment pattern of BVP with that of amnestic mild cognitive impairment (MCI) at high risk for conversion to Alzheimer’s disease (Gommeren et al., 2023; Bosmans et al., 2022). They found overlapping impairments in immediate memory and visuospatial cognition between the two groups, but delayed memory function was typically preserved in BVP patients (Gommeren et al., 2023; Bosmans et al., 2022). This pattern similarity suggests that vestibular loss may induce a cognitive impairment phenotype partially overlapping with early Alzheimer’s disease pathology, strongly supporting vestibular dysfunction as an independent modifiable risk factor for cognitive decline, particularly within the Alzheimer’s disease spectrum (Gommeren et al., 2023; Bosmans et al., 2022). Functional magnetic resonance imaging studies also indicate that BVL patients exhibit significantly lower activation levels in the right anterior hippocampus and parahippocampal regions during imagined standing or walking tasks compared to healthy controls (*p* < 0.001), suggesting that the absence of vestibular input leads to a specific attenuation of neural activity related to spatial navigation (Jahn et al., 2009). In summary, vestibular damage constitutes a multi-level evidence chain supporting its association with cognitive decline through structural atrophy, circuit dysfunction, and behavioral deficits. Animal studies further reveal subtle structural alterations: bilateral vestibular deafferentation (BVD) causes significant reduction in basal dendrite length in the CA1 region (*p* ≤ 0.0001) while apical dendrites remain intact. This suggests that vestibular input loss may disrupt neuronal information integration by selectively affecting specific dendritic subpopulations. In behavioral tests (e.g., radiant arm maze, Y-maze), vestibular-damaged animals exhibit persistent and specific spatial memory deficits, while non-spatial memory (e.g., novel object recognition) remains largely unaffected, highlighting the core role of the Vestibular-hippocampal pathway in spatial information processing (Balabhadrapatruni et al., 2016). Animal studies further supplement this evidence. The superior semicircular canal dehiscence (SSCD) model not only induces impaired decision-making ability (reduced d-prime values) but also shows significant correlation with alterations in cervical vestibular evoked myogenic potential (c + VEMP) amplitude, suggesting that vestibular dysfunction may be a potential cause of cognitive deficits (Mowery et al., 2023).

### 3.2 Effects of hippocampal degeneration on the vestibular system

The hippocampus and its connected entorhinal cortex form the core neural circuit hub for spatial memory and navigation, while also serving as a critical node for the advanced integration of vestibular information (Long and Zhang, 2021). Vestibular information is transmitted to the hippocampus via relay stations such as the vestibular nuclei, thalamus, and entorhinal cortex (Yun et al., 2025): The vestibular nuclei serve as primary relay stations, receiving peripheral vestibular signals and performing initial integration. These signals undergo sensory-cognitive conversion and filtering via the thalamus (primarily the ventromedial nucleus) before ultimately projecting to the hippocampus through the entorhinal cortex. The hippocampus, in turn, provides spatial contextual reference for vestibular signals through its position cell and grid cell systems, thereby supporting self-motion perception and path integration (Zwergal et al., 2024). When the hippocampus degenerates, the loss of spatial memory and contextual integration capabilities may prevent the vestibular system from accurately anchoring self-motion signals, leading to spatial disorientation (Anagnostou et al., 2018). This absence of spatial contextual reference may trigger “signal mismatch,” where vestibular afferent signals diverge from the spatial model anticipated by the hippocampus. Sepehry et al. (2024) noted in their traumatic brain injury (TBI) research that signal mismatch impairs the vestibular system’s ability to accurately integrate multimodal sensory inputs, leading to dizziness, balance disorders, and spatial disorientation (Zhu et al., 2024). This mechanism similarly applies to neurodegenerative diseases: disruption of the spatial reference framework due to hippocampal degeneration may exacerbate mismatches between vestibular signals and central expectations, creating a vicious cycle of vestibular dysfunction and cognitive decline.

Neurodegenerative diseases such as Alzheimer’s disease cause atrophy in multiple brain regions, including the hippocampus, entorhinal cortex, frontal lobe, and parietal lobe (Kang et al., 2024; Woodward et al., 2024; Oltmer et al., 2022). Atrophy in these areas may lead to various cognitive impairments, such as spatial cognition, executive function, attention, and language abilities (Kamil et al., 2018; Astillero-Lopez et al., 2022; Li et al., 2026; Lu et al., 2025). Among these, hippocampal degeneration may compromise its spatial information processing capacity, thereby affecting the regulation of vestibular function (Huang et al., 2023; Wei et al., 2019; Park and Kang, 2021). The hallmark pathological features of Alzheimer’s disease (AD)—β-amyloid (Aβ) plaques and neurofibrillary tangles (NFTs)—selectively involve the entorhinal cortex in the early stages (Oltmer et al., 2022; Kobro-Flatmoen et al., 2023). As the key gateway for vestibular information entering the hippocampus, neuronal loss and synaptic dysfunction in the entorhinal cortex may disrupt the transmission of vestibular signals to the hippocampus. This disruption could exacerbate spatial cognitive deficits, particularly impairing directional sense and navigational abilities (Huang et al., 2023; Vania et al., 2025; Wei et al., 2018). While other brain regions and pathways also contribute to spatial cognition, the Vestibular-hippocampal pathway likely plays a crucial role in maintaining directional sense and navigational capabilities.

Clinical studies indicate that vestibular-balance dysfunction exhibits a significant correlation with the severity of cognitive decline and demonstrates progressive deterioration within the Alzheimer’s disease (AD) spectrum. Clinical evidence indicates that during the preclinical stage of AD, vestibular impairment in patients with mild cognitive impairment (MCI) is already significantly higher than in age-matched healthy controls (Wei et al., 2019). This impairment worsens further after AD diagnosis: patients demonstrate markedly poorer postural stability across all testing conditions (e.g., eyes closed, soft foam surface) and exhibit greater anterior–posterior sway disturbance, indicating an objective, quantifiable deficit in the balance system’s ability to integrate multisensory information (Ashiri et al., 2021). Importantly, this impairment cannot be fully attributed to normal age-related multisystem decline. Studies reveal that AD patients exhibit specific difficulties in tasks requiring reliance on vestibular input and suppression of conflicting visual/proprioceptive information, compared to cognitively intact age-matched peers (Chepisheva, 2023). This progressive central sensory integration disorder directly translates into heightened fall risk, with annual fall incidence rates in AD patients reaching 2 to 3 times that of healthy elderly individuals (Chepisheva, 2023). The above evidence strongly suggests that pathological changes in AD (such as amyloid beta deposition and tau protein tangles) may disrupt vestibular information integration by affecting the hippocampus and associated multisensory integration cortices (e.g., posterior parietal cortex), leading to specific patterns of balance deficits. This review focuses on the “Vestibular-hippocampal” pathway in AD due to its pathological specificity and target circuitry. The hallmark pathology of AD, such as tau neurofibrillary tangles, early and preferentially affects the entorhinal cortex and hippocampus—structures central to integrating vestibular signals and forming spatial cognition (Chepisheva, 2023). Consequently, the progressive balance and spatial disorientation observed in AD patients can be directly traced to primary damage in this pathway, providing a pathological basis for distinguishing AD-specific impairments from universal functional changes associated with normal aging (Chepisheva, 2023). In contrast, the mechanisms underlying vestibular dysfunction in Parkinson’s disease (PD) differ, and a direct link to hippocampal degeneration remains unestablished. PD patients exhibit objective vestibular dysfunction (e.g., abnormal vestibular-evoked myogenic potentials), alongside hippocampal gray matter volume atrophy (Hawkins et al., 2021; Ferri et al., 2025). A key limitation, however, is that existing evidence reports these two abnormalities separately without establishing a causal chain linking “accumulating hippocampal pathology to vestibular dysfunction.” Current research suggests that vestibular dysfunction in PD patients (e.g., dizziness, postural instability) is more likely associated with brainstem involvement (e.g., vestibular nuclei, pontine tegmentum), cerebellar pathway impairment, or abnormalities in the basal ganglia-vestibular circuit (Gui et al., 2024). Within the “Vestibular-hippocampal” pathway framework central to this paper, PD serves as a valuable control model for elucidating how distinct initial pathologies produce similar balance disorder phenotypes. This rationalizes the present discussion’s focus on AD in terms of mechanistic depth.

In summary, vestibular damage not only directly causes hippocampal dysfunction and cognitive impairment through molecular, circuit, and structural alterations, but hippocampal degeneration can also conversely impair the processing and utilization of vestibular signals. This bidirectional relationship suggests that the Vestibular-hippocampal pathway may form a self-reinforcing vicious cycle in neurodegenerative diseases. Specifically, the absence of vestibular input diminishes the hippocampus’s capacity to integrate spatial information, leading to disrupted position cell firing, abnormal theta rhythms, and impaired synaptic plasticity, thereby exacerbating spatial memory and navigation deficits. Conversely, hippocampal degeneration, particularly early pathological involvement of the entorhinal cortex, disrupts critical stages of vestibular signal transmission and integration, increasing reliance on impaired self-motion perception and postural control. Clinically, this bidirectional mechanism manifests as concurrent spatial cognitive impairment and balance dysfunction in patients with Alzheimer’s disease, Parkinson’s disease, and various vestibular disorders (e.g., bilateral vestibular lesions, Meniere’s disease, and benign paroxysmal positional vertigo). This suggests both symptom categories may share a common neuropathological basis, often mutually reinforcing each other and accelerating disease progression. Although different vestibular disorders may influence cognitive function via the Vestibular-hippocampal pathway, their heterogeneous etiologies—such as the distinct mechanisms of endolymphatic hydrops in Meniere’s disease versus otolith dislodgement in BPPV—may limit the direct extrapolation of findings. Stratified analysis tailored to specific disease types is therefore necessary. This suggests that the Vestibular-hippocampal pathway is not merely a target of damage but a dynamically interacting vulnerable circuit in neurodegenerative diseases. The bidirectional vicious cycle within the Vestibular-hippocampal pathway not only offers a crucial perspective for understanding the pathomechanisms of neurodegenerative diseases but also provides novel targets for clinical intervention. Targeting any component of this cycle—such as vestibular rehabilitation training, cognitive training, or neuromodulation techniques—may yield dual benefits: improving vestibular function while delaying cognitive decline. This provides a theoretical basis for comprehensive treatment strategies in neurodegenerative diseases.

## 4 Clinical intervention evidence for bidirectional mechanisms

Intervention strategies targeting Vestibular-hippocampal pathway function aim to delay or reverse cognitive and balance decline in neurodegenerative diseases by improving peripheral vestibular input, enhancing central processing capacity, or modulating neural plasticity. This section systematically reviews the existing evidence and future potential of primary interventions, including vestibular rehabilitation training, cognitive training, and neuromodulation techniques.

### 4.1 Vestibular rehabilitation training

Vestibular Rehabilitation Therapy (VRT) is a personalized intervention based on physical therapy. It primarily enhances the compensatory capacity of the central nervous system—particularly the functional compensation of vestibular reflexes—through a series of specific exercises targeting the head, eyes, and trunk. As a multimodal intervention strategy, its core mechanism lies in repeatedly integrating visual, vestibular, and proprioceptive inputs through sensory-motor stimulation. This optimizes sensory weighting allocation and cross-modal reintegration capabilities, thereby promoting functional recovery in dynamic balance control, vertigo relief, and spatial perception. Standard VRT protocols typically incorporate multisensory balance training, including dynamic postural control, visual-vestibular conflict tasks, and gait adaptation exercises. These actively challenge and remodel central sensory integration pathways, accelerating overall recovery following vestibular system damage (Zhang S. et al., 2022; Vats et al., 2023; Loftin et al., 2020; Le Perf et al., 2025).

Basic research provides support for the neurobiological mechanisms underlying VRT. Animal studies indicate that sensorimotor rehabilitation promotes glial cell generation in the afferent vestibular nuclei, exerting neuroprotective effects and accelerating vestibular recovery (Marouane et al., 2021). Further research demonstrates that vestibular input activated during balance training directly modulates hippocampal neuroplasticity and enhances functional integration of the hippocampal-parietal cortex pathway. This suggests VRT’s effects extend beyond vestibular function itself, potentially influencing higher-order cognitive processes via the Vestibular-hippocampal pathway (Rogge et al., 2017).

Clinically, vestibular rehabilitation demonstrates positive outcomes for elderly populations and patients with mild cognitive impairment (MCI). For instance, in elderly and MCI patients with unilateral vestibular hypofunction (UVH), VRT significantly improves balance function and sensory reintegration abilities, thereby enhancing quality of life and independence in daily activities (Micarelli et al., 2018). Furthermore, functional imaging studies suggest VRT may exert its therapeutic effects by remodeling cognitive-related brain functional networks, providing evidence for its potential value in intervening neurodegenerative diseases (Chen et al., 2025).

In summary, vestibular rehabilitation not only alleviates motor and balance impairments by improving peripheral vestibular function but may also indirectly enhance cognitive abilities by modulating hippocampal plasticity and brain network connectivity. Thus, VRT serves as a comprehensive strategy bridging sensory-motor rehabilitation and cognitive intervention, offering new perspectives for exploring the “Vestibular-hippocampal pathway” in neurodegenerative disease management.

### 4.2 Cognitive training

Cognitive Training (CT) refers to an intervention strategy that employs systematic, structured cognitive task exercises to enhance or maintain specific cognitive domains such as memory, executive function, and spatial navigation (Trebbastoni et al., 2018; Maselli et al., 2019). Its theoretical foundation lies in brain plasticity—the principle that repeated practice promotes the activation and reorganization of relevant neural circuits, thereby improving impaired cognitive functions.

When exploring intervention strategies for the vestibular-hippocampal pathway, spatial navigation-related CT has emerged as a research focus due to its direct association with pathway function (Gamba et al., 2022). Recent studies have moved beyond general descriptions, developing specific interventions such as computerized spatial navigation training and virtual reality navigation tasks. These approaches provide concrete behavioral and neuroimaging evidence for the underlying mechanisms (Ellis et al., 2018; Pasquier et al., 2019).

For instance, computerized spatial navigation training has been employed as a tool for directionally activating hippocampal circuits. In a 14-day bed rest study simulating microgravity effects, Smith et al. used this training protocol to effectively mitigate activity-induced declines in spatial cognition, providing direct evidence for the plasticity of hippocampal-dependent navigational function (Marusic et al., 2018). Regarding training content design, research has distinguished between single-domain and multi-domain strategies. In animal models, single-domain training focuses on spatial navigation tasks (e.g., Morris water maze variants), while multi-domain training integrates spatial navigation, object recognition, and fear conditioning. The latter demonstrates more pronounced effects in improving the use of hippocampal-dependent spatial strategies and enhancing hippocampal volume (Mehla et al., 2023).

The mechanisms underlying spatial navigation-related cognitive training are closely linked to sensory integration and neural circuit remodeling. CT is thought to compensate for impaired vestibular input by modulating sensory weighting (Ellis et al., 2018). Computational modeling supports this view, suggesting that in patients with bilateral vestibular loss, cognitive training drives greater reliance on internal predictive models and visual cues, partially compensating for navigation deficits caused by vestibular input loss (Ellis et al., 2018).

In summary, specific spatial navigation cognitive training (e.g., computerized spatial navigation training) has demonstrated potential to compensate for insufficient vestibular input or neurodegenerative lesions by enhancing hippocampal circuit function and optimizing multisensory integration strategies. Although current research remains limited in sample size, protocol standardization, and long-term efficacy, these concrete examples provide actionable evidence-based foundations for interventions targeting the vestibular-hippocampal pathway. Future research should explore how such CT synergizes with vestibular rehabilitation training to develop integrated sensory-cognitive intervention models, thereby achieving more comprehensive functional improvements in the clinical management of neurodegenerative diseases.

### 4.3 Neuromodulation technology

In recent years, with deepening understanding of the role of the Vestibular-hippocampal pathway in neurodegenerative diseases, neurostimulation techniques based on vestibular stimulation have garnered increasing attention. Particularly, non-invasive methods such as galvanic vestibular stimulation (GVS) and noisy GVS have demonstrated potential in improving spatial cognition, motor coordination, and autonomic nervous system function in both basic and clinical research. Galvanic Vestibular Stimulation (GVS): A non-invasive technique that directly activates the vestibular system by applying electrical currents. Controlled currents delivered via electrodes attached to the mastoid bone selectively stimulate vestibular afferent pathways, mimicking natural vestibular input (Pasquier et al., 2019; Marchand et al., 2025).

Animal studies provide crucial evidence elucidating the neural mechanisms of vestibular stimulation. Research indicates that both direct electrical stimulation of vestibular receptors and passive whole-body rotation induce significant electrophysiological responses in the hippocampus. For instance, stimulating different vestibular receptors in rats generates triphasic local field potentials (LFPs) in both hippocampal hemispheres, demonstrating bilateral and hierarchical representation of vestibular input within the hippocampus (Hitier et al., 2021). Furthermore, rotational stimulation induces hippocampal theta rhythms and enhances long-term potentiation (LTP) by activating cholinergic septal-hippocampal pathways, suggesting vestibular input may directly promote hippocampal synaptic plasticity and spatial information processing (Tai et al., 2012). Microdialysis experiments further demonstrate that vestibular stimulation increases acetylcholine release in the hippocampus, providing molecular-level evidence for its role in enhancing learning and memory functions (Horii et al., 1994). In animal disease models, GVS demonstrates significant cognitive enhancement effects. For instance, in unilaterally labyrinthectomized (UL) mice, GVS accelerates recovery of short- and long-term spatial memory, navigational function, and locomotor abilities (Nguyen et al., 2021). These findings suggest that vestibular stimulation not only compensates for peripheral vestibular deficits but also promotes learning and memory recovery by enhancing hippocampal neural circuit activity.

In human studies, functional imaging results indicate that vestibular stimulation activates the hippocampus and parahippocampal cortex, consistent with the subjective sensations of self-motion and spatial disorientation experienced during stimulation (Vitte et al., 1996). Clinical behavioral studies further demonstrate that subthreshold noise vestibular stimulation improves memory and cognitive performance in some individuals, suggesting its potential to modulate central cognitive networks (Smith et al., 2010).

Among neurodegenerative disease populations, noise GVS has been explored as an adjunctive therapeutic approach. Studies demonstrate that this method can improve bradykinesia and autonomic reactivity in patients with Parkinson’s disease and multiple system atrophy, while also reducing reaction times in certain cognitive tasks (Pasquier et al., 2019; Pan et al., 2008). These findings provide preliminary support for the clinical translation of GVS, particularly highlighting its unique advantages in addressing the dual motor-cognitive deficits.

In summary, vestibular electrical stimulation as a neuromodulation technique offers novel insights for improving cognitive and motor impairments in neurodegenerative diseases. However, translating it into a stable and reliable clinical intervention remains challenging. Current studies exhibit significant variability in stimulation parameters (e.g., current intensity, waveform, frequency, treatment duration), and most human investigations feature small sample sizes and exploratory designs. For instance, current intensities range from 0.1 mA to 0.7 mA across existing literature, while frequencies span from direct current to random noise (<30 Hz) (Adel Ghahraman et al., 2016; Wuehr et al., 2024). No consensus has been established regarding the optimal combination of parameters (dosage) and treatment frequency to achieve clinically significant efficacy. Future research requires rigorously designed, large-scale randomized controlled trials to systematically optimize stimulation parameters. Integrating precise electrophysiological monitoring with imaging assessments will further elucidate the underlying mechanisms and clarify the precise therapeutic effects on cognitive and vestibular function in neurodegenerative disease patients, thereby advancing this technology from experimental exploration to clinical practice.

## 5 Discussion

This paper systematically reviews the bidirectional vicious cycle mechanism of the Vestibular-hippocampal pathway in neurodegenerative diseases such as Alzheimer’s disease (AD). At the level of basic anatomy and neural pathways, it systematically elucidates the organizational patterns of peripheral and central structures within the vestibular system and their signal transmission pathways, while also detailing the functional subdivisions of the hippocampal structure and its central role in spatial cognition. Furthermore, by mapping multisynaptic networks including the vestibular-thalamic-cortical pathway, theta rhythm pathway, head direction pathway, and vestibular-cerebellar-cortical pathway, the neural basis for vestibular information input to the hippocampus and its regulation of spatial memory and navigation functions is established. At the molecular level, vestibular dysfunction induces neuroinflammation, dysregulation of neurotrophic factors, and abnormalities in multiple receptor/neurotransmitter systems, thereby impairing hippocampal synaptic plasticity and spatial memory encoding capacity. At the circuit level, vestibular input loss disrupts position cell firing, causes theta rhythm abnormalities, and reduces spatial representation stability, further undermining the neural network foundation of spatial cognition. Ultimately, at the structural and behavioral levels, this not only causes hippocampal atrophy and spatial navigation deficits but also exacerbates balance disorders and fall risks by weakening the vestibular system’s regulatory role in motor and cognitive functions. Conversely, early pathological changes in the hippocampus and entorhinal cortex (such as Aβ deposition and neurofibrillary tangles) impair vestibular information integration and contextual referencing, creating a self-reinforcing vicious cycle. Regarding intervention strategies, this paper evaluates multi-level approaches including vestibular rehabilitation training, cognitive training, and neuromodulation techniques (such as electrical vestibular stimulation). These strategies demonstrate potential to delay the co-degeneration of cognitive and vestibular functions by enhancing neuroplasticity and optimizing sensory integration and regulatory circuitry. This multi-level, bidirectionally interactive mechanism framework provides an integrated perspective for understanding the co-occurring vestibular and cognitive impairments in neurodegenerative diseases.

Although current evidence suggests that the Vestibular-hippocampal pathway plays a crucial role in the progression of neurodegenerative diseases, its clinical translation still faces significant challenges. 1. First, mechanism studies in this field rely heavily on animal models (such as Sprague–Dawley rats with vestibular lesions and APP/PS1 double transgenic mice as AD models). However, significant differences exist between animals and humans in vestibular-dependent behaviors, cognitive assessment methods, and the temporal progression of pathological development. These discrepancies limit the validity of directly extrapolating findings from animal studies to human mechanisms. 2. Second, clinical evidence predominantly consists of cross-sectional association studies, making it difficult to establish causality between vestibular dysfunction and cognitive decline. Whether vestibular abnormalities represent a cause, consequence, or shared pathological manifestation of neurodegeneration requires further clarification through prospective longitudinal studies and causal intervention trials. Furthermore, developing quantitative vestibular assessments (e.g., vestibular evoked myogenic potentials, video head pulse testing) or vestibular-related functional imaging markers into tools for early diagnosis or staging of neurodegenerative diseases requires standardized protocols and large-scale validation. On the other hand, intervention strategies targeting this pathway demonstrate potential translational value: vestibular rehabilitation training and cognitive training can improve compensation and neuroplasticity; neuromodulation techniques (such as noise vestibular electrical stimulation) have shown in animal models to enhance hippocampal theta rhythms and cholinergic transmission, thereby improving spatial learning. However, efficacy parameters, suitable patient populations, and long-term effects in human patients require systematic investigation.

Several critical gaps remain in this field. Future research should focus on: First, developing animal models that simultaneously mimic vestibular dysfunction and neurodegenerative pathology (e.g., combined vestibular injury and transgenic models) to elucidate specific molecular pathways and time-dependent interactions in bidirectional communication. Second, employing multimodal imaging and electrophysiological techniques to longitudinally track the dynamic relationship between vestibular function and hippocampal structure–function changes in patient cohorts, supplemented by pathological validation using autopsy samples to enhance the reliability of clinical mechanism inferences; Third, exploring translational-potential specific molecular targets within the Vestibular-hippocampal pathway (e.g., glial cell activation pathways or synaptic receptor balance) and investigating their regulatory strategies; Fourth, optimize neurostimulation strategies based on vestibular stimulation, such as through individualized stimulation parameters, closed-loop feedback systems, or integration with other cognitive training, to enhance intervention efficacy. Additionally, examining interactions between other sensory systems (e.g., olfactory, visual) and the Vestibular-hippocampal pathway may offer new insights into multisensory compensatory mechanisms in neurodegenerative diseases.

In summary, the Vestibular-hippocampal pathway serves as a critical bridge integrating sensory input and cognitive function. Its bidirectional deterioration mechanism offers a novel perspective for understanding the complex pathology of neurodegenerative diseases. By elucidating this pathway’s pivotal role in disease progression, we not only advance our understanding of the multidimensional co-occurrence of symptoms in neurodegenerative diseases but also establish theoretical foundations and pathways for developing early biomarkers and novel intervention strategies. Future research should adopt interdisciplinary approaches to integrate evidence across molecular, circuit, and behavioral levels, thereby advancing clinical translation in this field.
